# Hospital survey on patient safety culture in China

**DOI:** 10.1186/1472-6963-13-228

**Published:** 2013-06-24

**Authors:** Yanli Nie, Xuanyue Mao, Hao Cui, Shenghong He, Jing Li, Mingming Zhang

**Affiliations:** 1 Chinese Evidence-based Medicine Center, West China Hospital, Sichuan University, Chengdu, 610041, PR China; 2 School of Public Health, Sichuan University, Chengdu, PR China; 3 Department of Nursing, Yijishan Hospital, Wannan Medical College, Wuhu, PR China

**Keywords:** Patient Safety Culture, Hospital Questionnaire, Health Care Workers, Positive Response

## Abstract

**Background:**

Patient safety culture is an important measure in assessing the quality of health care. There is a growing recognition of the need to establish a culture of hospital focused on patient safety. This study explores the attitudes and perceptions of patient safety culture for health care workers in China by using a Hospital Survey on Patient Safety Culture (HSPSC) questionnaire and comparing it with the psychometric properties of an adapted translation of the HSPSC in Chinese hospitals with that of the US.

**Method:**

We used the modified HSPSC questionnaire to measure 10 dimensions of patient safety culture from 32 hospitals in 15 cities all across China. The questionnaire included 1160 Chinese health-care workers who consisted of predominately internal physicians and nurses. We used SPSS 17.0 and Microsoft Excel 2007 to conduct the statistical analysis on survey data including descriptive statistics and validity and reliability of survey. All data was input and checked by two investigators independently.

**Result:**

A total of 1500 questionnaires were distributed of which 1160 were responded validly (response rate 77%). The positive response rate for each item ranged from 36% to 89%. The positive response rate on 5 dimensions (*Teamwork Within Units, Organization Learning-Continuous Improvement, Communication Openness, Non-punitive Response and Teamwork Across Units*) was higher than that of AHRQ data (P < 0.05). There was a statistical difference on the perception of patient safety culture in groups of different work units, positions and qualification levels. The internal consistency of the total survey was comparatively satisfied (Cronbach’s α = 0.84).

**Conclusion:**

The results show that amongst the health care workers surveyed in China there was a positive attitude towards the patient safety culture within their organizations. The differences between China and the US in patient safety culture suggests that cultural uniqueness should be taken into consideration whenever safety culture measurement tools are applied in different culture settings.

## Background

Patient safety is an important component of health care quality. Patient safety, including the measurement of patient safety culture is a top priority in developed countries today [1]. Research shows that safety and efficient care requires all the various elements of a health care system be well integrated and coordinated [2,3].

Patient safety in the context of health care organizations was highlighted following the Institute of Medicine (IOM) report *“To Error is Human: Building a Safer Health System”*[4]. This report argued for a safety culture in which adverse events can be reported without people being blamed, and that when mistakes occur that lessons are learned. Therefore, if hospitals want to improve patient safety, it is important to know more about the views of their staff in relation to the culture of patient safety.

Patient safety culture, also referred to patient safety climate, is the overall behavior of individuals and organizations, based on a common set of beliefs and values that are aimed at reducing the opportunities for patient harm [5,6]. Related research shows that when a positive patient safety culture exists, it will promote patient safety and help to improve patient safety standards, including the capacity and willingness to report minor errors, self-reporting errors, safety behaviors and safety audit rating [7-9].

To date, many developed countries have initiated the research into the role played by patient safety culture research. On a global basis, several international organizations promote the establishment of a culture of patient safety: the World Alliance for Patient Safety, the National Patient Safety Agency in the UK, the Agency for Healthcare Research and Quality in US and the Australia Commission of Safety and Quality in Australia. Related studies have also been conducted in Asian [10,11]. Most of the studies focus on evaluation of the psychometric properties of the HSPSC which has been translated in their own languages (Japan, Norway, Turkey, Netherlands, etc.) [11-14]. The studies from Japan and Norway showed the internal reliability of the subscale scores vary between factors, ranging from 0.46 to 0.88, of which factor ‘stuffing’ had the lowest reliability (0.46 and 0.59 respectively) [11,12]. The internal consistency of some items in Arabic version of the HSOPSC was lower than that of the original items in the US study [15]. While there was some evidence suggested that the translation of the HSOPSC was acceptable in reliability and good construct validity [11,14,16]. The studies by Belgium, Turkey and Taiwan also used HSPSC to measure patient safety culture in their own countries[10,13,17].

In our research, we analyzed the attitudes and experiences of patient safety culture that Chinese health care workers had using a modified version of Hospital Survey of Patient Safety Culture (HSOPSC) of AHRQ. The purpose of this study was to measure the patient safety culture in China’s hospitals and discuss some of the phenomena unique to China. We also compare some of the findings with existing data from benchmark scores using HSOPSC. Meanwhile, we intend to assess the quality of this investigative questionnaire. The findings of this study will provide health care organizations a better understanding about hospital culture and the extent to which patient safety attitudes are present in China.

## Methods

### Questionnaire

The questionnaire was translated and modified to suit the Chinese system. HSOPSC (the original U.S. English version 2010) was developed by Agency for Healthcare Research and Quality (AHRQ) in 2004 [18]. The original questionnaire of 2010 version was designed to assess 12 dimensions of health care with 42 items of patient safety culture. But two dimensions (Frequency of Events Reported and Handoffs and Transitions) with 13 items were omitted due to being sensitive, not adequately providing a response or semantically redundant or ambiguous because of translation [19]. Finally, 10 dimensions containing 29 items of hospital survey on patient safety culture were adopted (Table 1).

**Table 1:** Demographic characteristic of respondents

***Characteristics***	***Physicians (n = 301)***	***Nurses (n = 722)***	***Others (n = 137)***	***Total (n = 1160)***
Working units				
Internal medicine	79(23.1)	234(68.2)	30(8.7)	343(100 ^a^ )
Surgery department	106(27.4)	250(64.8)	30(7.8)	386(100)
Other units	116(26.9)	138(52.4)	77(17.7)	431(100)
Years in hospital				
< 1	55(17.5)	192(61.1)	67(21.3)	314(100)
1-5	89(19.6)	336(74.0)	29(6.4)	454(100)
6-10	44(27.3)	108(67.1)	9(5.6)	161(100)
≥11	113(48.9)	86(37.2)	32(13.9)	231(100)
Years in department				
< 1	64(16.9)	243(64.1)	72(19.0)	379(100)
1-5	103(20.6)	365(73.2)	31(6.2)	499(100)
6-10	49(37.1)	74(56.1)	9(6.8)	132(100)
≥11	85(56.7)	40(26.7)	25(16.6)	150(100)
Hours working per week				
<20	11(42.3)	7(26.9)	8(30.8)	26(100)
20-39	31(15.7)	129(65.5)	37(18.8)	197(100)
40-59	136(18.3)	531(71.5)	76(10.2)	743(100)
≥60	123(63.4)	55(28.4)	14(8.2)	194(100)
Contact with patient directly				
Yes, often	294(26.8)	702(64.1)	99(9.0)	1095(100)
No	6(9.5)	19(30.2)	38(60.3)	63(100)

^a^ Parenthesis represent percentage.

All items of HSOPSC questionnaire were developed based on the 5-point Likert response scale of agreement (“Strongly disagree” to “Strongly agree”) or frequency (“Never” to “Always”).

Two medical students translated the HSOPSC questionnaire into Chinese with a background in patient safety. The translation was double checked and reviewed by another two professors with background in patient safety, medicine and English.

### Sample

This questionnaire was conducted in July to December in 2011 involving 1160 healthcare workers including physicians (surgical clinicians and internal clinicians) from each department and nurses representing different nursing units. Convenient sampling was used to select hospitals and participants including 32 hospitals in 15 cities across China. We included health care workers who are staff working in hospitals. We also included any kind of hospitals including specialized hospitals and traditional medicine hospitals.

Permission to conduct the investigation was granted by the hospitals or departments. The participants were informed of the purpose of the survey and voluntarily completed a paper copy of the questionnaire anonymously by the investigators who were present. The participants were encouraged to ask any questions if they did not understand the questionnaire. Questionnaires were regarded as invalid ones if there were inconsistent answers (e.g., an item with more than one answer). The questionnaires were valid if at least 70% of items were completed.

### Data analysis

#### Data collection

After receiving the completed questionnaires, a preprocessing step was applied to remove incomplete or invalid data and based on the study by Hellings J [17]. The exclusion criteria were similar to the two studies [17,20]. They were as follows: 1) there was no entire section completed; 2) there was fewer than half items answered; or all the items answered the same. All data was entered by three researchers (Nie YL, Cui H, and SH H) independently, and then were cross-checked mutually by Epidata (version, 3.02). In case of doubts or disagreement in some answers, we looked into the original questionnaires. Negatively worded items were reversed to ensure that positive answers indicated a higher score. Most of the items in the questionnaire used the Likert 5- point response scale of agreement (*Strongly disagree* to *Strongly agree*) or frequency (*Never* to *Always*),so the lowest three scoring (1–3) answers (*Strongly disagree/Disagree/Neither Agree nor Disagree* or *Never/Rarely/Sometimes* or *Failing/Poor/Acceptable*), the highest two scoring (4–5) answers (*Agree/Strongly agree* or *Most of the time/Always* or *Very good/Excellent*) [21], as well as the highest two scoring answers were perceived as positive response answers, and the lowest three scoring answers were deemed other response answer. We calculated the positive response rate to analyze the positive attitudes towards patient safety culture among different populations according to the formula by Grant MJ [22].

### Descriptive statistics

We analyzed the demographic characteristics of the respondents with the Excel 2003. The number of positive response / positive response rates of all the items were also summarized. Positive response rate was used to evaluate the attitudes towards patient safety culture on different dimensions or items.

We used the Chi-Square test to analyze whether there was a statistical difference on health care workers in different sections, professionals and qualification levels towards patient safety culture. We used the Kruskal-Wallis test to infer if there was a statistical difference on *Patient Safety Grade* and *Number of Events Reported* in Chinese hospitals compared with that of US hospitals, with the significant level of P = 0.05 [21].

We calculated the reliability and exploration factor analysis to evaluate the quality of the questionnaire. Internal consistency value (Gronbach’s α ≥ 0.70) for newly developed scales was recommended. Structure validity was explored using principal component factor analysis by Kaiser-Meyer-Olkin Measure of Sampling Adequacy (KMO > 0.7) and by Bartlett’s Test of Sphericity *P* < 0.05.

### Ethic

The study was conducted according to the principle of Helsinki Declaration. The protocol has been reviewed and approved by the Chinese Ethic Committee of Registering Clinical Trials (ChiECRCT-2011021).

## Results

### Sample and response statistics

A total of 1500 questionnaires were distributed of which 1160 were responded validly (response rate 77%). Seven hundred and twenty two (66%) of the respondents were nurses, 386 (33%) were surgical clinicians and 343 (30%) were internal medicine clinicians. The majority of respondents (94%) usually dealt with patients directly (Table 1).

Table 2 showed that the positive response rate for the 10 patient safety culture dimensions ranged from 45% to 88%, the mean positive response rate was 65%. The lowest positive response rate of dimension was *Staffing* (45%), while the highest positive response rate of dimension was O*rganization Learning-Continuous Improvement* (88%). There were three dimensions of which positive response rate were less than 60% such as *Overall Perception of Patient Safety* (55%), *Feedback & Communication About Error* (50%), and *Staffing* (45%). The positive response rate for the rest of the items ranged from 36% to 89%. The highest positive response rate of the items was *After we make changes to improve patient safety, we evaluate their effectiveness* (89%), while the lowest positive response rate of the item was *Whenever pressure builds up, my supervisor/manager wants us to work faster, even if it means taking shortcuts* (36%).

**Table 2:** Positive response rate of each item and Cronbach’s α for dimensions

**Dimension/items(internal consistency reliability coefficient)**	**US**	**China**
**1.Teamwork Within Units (**Cranach’s α = 0.72**)**	80%	84%
A1. People support one another in this facility	86%	87%
A3. When a lot of work needs to be done quickly, we work together as a team to get the work done	86%	87%
A4. In facility, people treat each other with respect	78%	80%
A11. When one area in this unit gets really busy, others help out	69%	81%
**2.Supervisor/Manager Expectations & Actions Promoting Patient Safety (**Cranach’s α = 0.51**)**	75%	63%
B2. Manager says a good word when he/she sees a job done according to established	76%	76%
B3. Whenever pressure builds up, my supervisor/manager wants us to work faster, even if it means taking shortcuts	74%	36%
B4. My supervisor/manager overlooks patient safety problems that happen over and over	76%	78%
**3.Organizational Learning—Continuous Improvement (**Cranach’s α = 0.74**)**	72%	88%
A6. We are actively doing things to improve patient safety.	84%	87%
A13. After we make changes to improve patient safety, we evaluate their effectiveness.	69%	89%
**4.Management Support for Patient Safety (**Cranach’s α = 0.67**)**	72%	69%
F1. Hospital management provides a work climate that promotes patient safety.	81%	71%
F8. The actions of hospital management show that patient safety is a top priority.	75%	70%
F9. Hospital management seems interested in patient safety only after an adverse event happens	61%	65%
**5.Overall Perceptions of Patient Safety (**Cranach’s α = 0.64**)**	66%	55%
A10. It is just by chance that more serious mistakes don't happen around here.	62%	61%
A17. We had patient safety problems in this unit.	64%	37%
A18. Our procedures and systems are good at preventing errors from happening.	72%	65%
**6.Feedback & Communication About Error (**Cranach’s α = 0.64**)**	64%	50%
C1. We are given feedback about changes put into place based on event reports.	56%	54%
C3. We are informed about errors that happen in this unit.	65%	64%
C5. In this unit, we discuss ways to prevent errors from happening again.	72%	53%
**7 Communication Openness (**Cranach’s α = -0.47**)**	62%	65%
C2. Staff will freely speak up if they see something that may negatively affect patient care.	75%	51%
C4. Staffs are afraid to ask questions when something does not seem right.	47%	80%
C6. Staffs feel free to question the decisions or actions of those with more authority.	63%	64%
**8.Nonpunitive Response to Errors (**Cranach’s α = 0.75**)**	44%	60%
A8. Staff feel like their mistakes are held against them.	50%	53%
A12. When an event is reported, it feels like the person is being written up, not the problem.	46%	67%
A16. Staff worry that mistakes they make are kept in their personnel file.	35%	60%
**9 Teamwork Across Units (**Cranach’s α = -0.63**)**	58%	66%
F4. There is good cooperation among hospital units that need to work together.	60%	66%
**10. Staffing (**Cranach’s α = 0.63**)**	56%	45%
A2. We have enough staff to handle the workload.	56%	42%
A5. Staffs in this unit work longer hours than is best for patient care.	51%	38%
A7. We use more agency/temporary staff than is best for patient care.	44%	37%
A14. We work in “crisis mode” trying to do too much, too quickly.	45%	61%

However, *Hospital Survey on Patient Safety Culture-2012 User Comparative Database Report*[21] showed that the average positive response rate of 12 dimensions ranged from 35% to 86%, the overall average positive response rate for dimensions was 63%. The lowest positive response rate of item was *Staff worry that mistakes they make are kept in their personal file* (35%) and the highest positive response rate items were *People support one another in this facility* (86%) and *When a lot of work needs to be done quickly, we work together as a team to get the work done* (86%). There were 11 items of which the positive response rate were less than 60%, see details in Table 2.

There were some differences between the adapted Chinese HSPSC with that of original US HSPSC, so only the same items were compared to explore the differences of perceptions towards patient safety culture between the two countries. The results showed that there was a significant difference on 10 items (P < 0.05), of which the positive response rate on 10 items in China was higher than that of the US. These dimensions were (1) *Teamwork Within Units, Organization (2) Learning-Continuous Improvement, (3) Communication Openness, (4) Nonpunitive response and (5) Teamwork Across Units*. However, there was a significant difference on 10 items (P < 0.05), which of the positive response rate on 10 items in China was lower than that of the US, these dimensions were (1) *Supervisor/Manager Expectations & Actions Promoting (2) Patient Safety, Management Support for Patient Safety, (3) Overall Perception of Patient safety*, (3) *Feedback & Communication About Errors and (4) Staffing* (Table 2).

### Comparative results

The results showed that there was a significant difference on eight dimensions between physicians and nurses (i.e. *Teamwork Within Units, Organization Learning-Continuous Improvement, Management Support for Patient Safety, Feedback & Communication About Errors, Overall Perception of Patient Safety, Communication Openness, Non-punitive response to Errors* and *Staffing*, P < 0.05). The positive response rate of two items of nurses was lower than that of physicians (*It is just by chance that more serious mistakes don’t happen around here* and *Staff will freely speak up if they see something that may negatively affect patient care,* P < 0.05*)*. The positive response rate of other items of nurses was higher than that of physicians (Table 3).

**Table 3:** The comparison of attitudes of nurse versus physicians on patient safety culture

**Items**	***Nurse***		***Physicians***		**χ**^**2**^	**p**
	**NPR**	**NOR**	**NPR**	**NOR**		
A1. People support one another in this facility	624	97	270	31	1.93	0.17
A3. When a lot of work needs to be done quickly, we work together as a team to get the work done	647	75	250	51	8.45	0.01
A4. In facility, people treat each other with respect	692	29	267	34	19.42	0.01
A11. When one area in this unit gets really busy, others help out	607	115	231	70	7.7	0.01
B2. Manager says a good word when he/she sees a job done according to established	540	182	242	59	3.78	0.06
B3. Whenever pressure builds up, my supervisor/manager wants us to work faster, even if it means taking shortcuts	458	264	203	98	1.49	0.22
B4. My supervisor/manager overlooks patient safety problems that happen over and over	559	163	243	58	1.37	0.24
A6. We are actively doing things to improve patient safety.	646	75	250	51	8.41	0.01
A13. After we make changes to improve patient safety, we evaluate their effectiveness.	654	68	258	43	5.2	0.02
F1. Hospital management provides a work climate that promotes patient safety.	536	186	193	108	10.62	0.01
F8. The actions of hospital management show that patient safety is a top priority.	590	132	233	68	2.51	0.11
F9. Hospital management seems interested in patient safety only after an adverse event happens	478	244	193	108	0.41	0.52
A10. It is just by chance that more serious mistakes don't happen around here.	556	166	271	30	23.27	0.01
A17. We had patient safety problems in this unit.	446	276	195	106	0.82	0.36
A18. Our procedures and systems are good at preventing errors from happening.	497	225	174	127	11.45	0.01
C1. We are given feedback about changes put into place based on event reports.	385	337	169	132	0.68	0.41
C3. We are informed about errors that happen in this unit.	490	232	163	139	17.31	0.01
C5. In this unit, we discuss ways to prevent errors from happening again.	419	303	136	165	14.13	0.01
C2. Staff will freely speak up if they see something that may negatively affect patient care.	347	375	175	126	8.63	0.01
C4. Staffs are afraid to ask questions when something does not seem right.	596	126	224	77	8.83	0.01
C6. Staffs feel free to question the decisions or actions of those with more authority.	593	129	232	69	3.48	0.06
A8. Staff feel like their mistakes are held against them.	646	75	237	64	21.74	0.01
A12. When an event is reported, it feels like the person is being written up, not the problem.	615	107	250	51	0.73	0.39
A16. Staff worry that mistakes they make are kept in their personnel file.	644	75	273	28	0.29	0.59
F4. There is good cooperation among hospital units that need to work together.	480	242	184	117	2.67	0.10
A2. We have enough staff to handle the workload.	457	265	146	155	19.21	0.01
A5. Staffs in this unit work longer hours than is best for patient care.	450	272	182	118	0.25	0.81
A7. We use more agency/temporary staff than is best for patient care.	489	233	171	130	11.06	0.01
A14. We work in “crisis mode” trying to do too much, too quickly.	606	116	250	51	0.12	0.79

Legend:*NPR*, Number of positive response answers; *NOR*, Number of other response answers.

Incidence of patient safety events was closely related to the qualification level of physicians. Our result showed that there was a significant difference in the positive response rate on seven dimensions (i.e. *Teamwork Within Units, Management Support for Patient Safety, Teamwork Across Units, Feedback & Communication About Errors, Overall Perception of Patient Safety, Communication Openness* and *Non-punitive Response to Errors*, P < 0.05) for residents, attending physicians, deputy directors and chief physicians. Furthermore, the positive response rate of physicians with high qualification (chief physicians) on two dimensions (*Overall Perception of Patient safety* and *Feedback & Communication About Errors*) was higher than those having a low qualification level (residents), while the positive response rate of healthcare professionals with a high qualification level on five dimensions (*Teamwork Within Units, Management Support for Patient Safety, Communication Openness, Teamwork Across Units* and *Non-punitive Response to Errors*) was lower than that of those who have low qualification levels (Table 4).

**Table 4:** The attitudes of physicians with different levels on patient safety culture

**Items**	**Residents**		**Attending physicians**		**Deputy directors**		**Chief physicians**		***χ***^***2***^	***P***
	**NPR**	**NOR**	**NPR**	**NOR**	**NPR**	**NOR**	**NPR**	**NOR**		
A1. People support one another in this facility	98	10	77	8	57	7	38	6	0.76	0.86
A3. When a lot of work needs to be done quickly, we work together as a team to get the work done	98	10	69	16	44	20	39	5	14.98	0.01
A4. In facility, people treat each other with respect	99	9	72	13	58	6	38	6	2.77	0.43
A11. When one area in this unit gets really busy, others help out	88	20	60	25	49	15	34	10	3.16	0.37
B2. Manager says a good word when he/she sees a job done according to established	95	13	67	18	47	17	33	11	6.81	0.08
B3. Whenever pressure builds up, my supervisor/manager wants us to work faster, even if it means taking shortcuts	66	42	56	29	47	17	34	10	5.03	0.17
B4. My supervisor/manager overlooks patient safety problems that happen over and over	95	13	66	19	49	15	33	11	5.78	0.12
A6. We are actively doing things to improve patient safety.	93	15	70	15	49	15	38	6	3.00	0.39
A13. After we make changes to improve patient safety, we evaluate their effectiveness.	95	13	69	16	55	9	39	5	2.18	0.54
F1. Hospital management provides a work climate that promotes patient safety.	84	24	42	43	40	24	27	17	16.91	0.01
F8. The actions of hospital management show that patient safety is a top priority.	90	18	61	24	46	18	36	8	5.31	0.15
F9. Hospital management seems interested in patient safety only after an adverse event happens	77	31	51	34	38	26	27	17	3.80	0.28
A10. It is just by chance that more serious mistakes don't happen around here.	96	12	77	8	57	7	41	3	0.74	0.86
A17. We had patient safety problems in this unit.	50	58	64	21	45	19	36	8	26.66	0.01
A18. Our procedures and systems are good at preventing errors from happening.	76	32	40	45	38	26	20	24	13.79	0.01
C1. We are given feedback about changes put into place based on event reports.	69	39	46	39	32	32	22	22	4.41	0.22
C3. We are informed about errors that happen in this unit.	64	44	45	40	30	34	24	20	2.54	0.47
C5. In this unit, we discuss ways to prevent errors from happening again.	47	61	40	45	46	18	32	12	20.78	0.01
C2. Staff will freely speak up if they see something that may negatively affect patient care.	76	32	50	35	29	35	20	24	13.84	0.01
C4. Staffs are afraid to ask questions when something does not seem right.	83	25	64	21	47	17	30	14	1.30	0.73
C6. Staffs feel free to question the decisions or actions of those with more authority.	78	30	68	17	55	9	31	13	5.77	0.12
A8. Staff feel like their mistakes are held against them.	92	16	66	19	45	19	34	10	5.49	0.14
A12. When an event is reported, it feels like the person is being written up, not the problem.	99	9	69	16	47	17	35	9	10.46	0.02
A16. Staff worry that mistakes they make are kept in their personnel file.	99	9	77	8	57	7	40	4	0.33	0.96
F4. There is good cooperation among hospital units that need to work together.	85	23	44	41	32	32	23	21	21.89	0.01
A2. We have enough staff to handle the workload.	65	43	36	49	35	29	19	25	7.56	0.06
A5. Staffs in this unit work longer hours than is best for patient care.	64	43	49	36	38	26	31	13	2.16	0.54
A7. We use more agency/temporary staff than is best for patient care.	66	42	45	40	40	24	20	24	4.47	0.21
A14. We work in "crisis mode" trying to do too much, too quickly.	90	18	67	18	53	11	40	4	3.01	0.39

Legend:*NPR*, Number of positive response answers; *NOR*, Number of other response answers.

### Patient safety grade/number of events reported both in China and the US

According to the *Hospital Survey on Patient Safety Culture-2012 User Comparative Database Report*, the positive response rate on *Patient Safety Grade* in the US was 75%, while it was 73% in China. There was no significant difference between the two groups (P = 0.223, see Table 2). However, there were significant differences on all the answers of *Patient Safety Grade and the Number of Events Reported* between the two countries (Table 5).

**Table 5:** The comparisons of patient safety grade between different professionals

**Patient safety grade**	**Physicians (%)**	**Nurses (%)**	**Others (%)**	**Overall (%)**	**Benchmark (%)**
Excellent	55^a^(18)	122(17)	23(12)	17	30
Very good	157(52)	420(58)	75(12)	56	45
Acceptable	79(26)	163(23)	35(13)	24	20
Pool	10(3)	15(2)	4(14)	3	4
Failing	0(0)	2(0.3)	0(0)	0.2	1

^a^ number of the respondents.

### Reliability and validity

The reliability of the 10 dimensions was shown in Table 2. For the 10 dimensions, the internal consistency (Cronbach’s α) ranged from 0.40 to 0.64, and the Cronbach’s α of the total scale in our study was 0.84 which was lower than that of the original factor scale in the US.

Table 6 displayed the inter-correlations of the 10 dimensions, and correlations between the scale scores were also calculated. *Management Support for Patient Safety* and *Overall Perception of Patient Safety* (r = 0.77) are most correlated*,* while *Teamwork Within Units and Communication Opennes*s (r = 0.10) were least correlated. The highest correlation was 0.65 between *Feedback & Communication about Errors* and the scale (r = 0.65), and the correlation between each dimension and the total scale is significantly different (Table 6).

**Table 6:** Correlation with the total scale and inter-correlations of the 10 dimensions

**Dimensions**	**1**	**2**	**3**	**4**	**5**	**6**	**7**	**8**	**9**	**10**	**Total**
1. Teamwork Within Units	1.00	0.63	0.37	0.33	0.31	0.28	0.10	0.05	0.24	0.27	0.52^*^
2. Supervisor/Manager Expectations & Actions Promoting Patient Safety		1.00	0.32	0.44	0.55	0.48	0.19	0.13	0.42	0.43	0.64^*^
3. Organizational Learning—Continuous Improvement			1.00	0.70	0.39	0.31	0.07	−0.04	0.20	0.32	0.41^*^
4. Management Support for Patient Safety				1.00	0.77	0.47	0.16	0.08	0.40	0.46	0.53^*^
5. Overall Perceptions of Patient Safety					1.00	0.72	0.25	0.15	0.47	0.53	0.63^*^
6. Feedback & Communication About Error								0.18	0.42	0.43	0.65^*^
7. Communication Openness								0.67	0.26	0.14	0.48^*^
8. Nonpunitive Response to Errors								1.00	0.57	0.09	0.40^*^
9. Teamwork Across Units									1.00	0.56	0.59^*^
10. Staffing										1.00	0.60^*^

^*^All correlations are significant at P < 0.001.

Bartlett’s test of the 29 items on patient safety culture demonstrated a sufficient inter-item correlation: χ^2^ = 2163.578, df = 1159, P < 0.01. Furthermore, the Kaiser –Meyer-Olkin measure of sampling adequacy was satisfactory, with a value of 0.829. Explorative factor analysis was performed using principal component analysis with varimax rotation. Using the explorative factor analysis drew eight factors. The factors cumulatively explained 60 % of the variance in the survey and the result was acceptable.

Table 7 demonstrated the factor loadings for each item (all loadings > 0.40). Factor one loadings on five dimensions (*Teamwork Within Units, Management Support for Patient Safety, Overall Perceptions of Patient Safety, Communication Openness* and *Teamwork Across Units*), and factor two and six loading on *Supervisor/ManagerExpectations & Actions Promoting Patient Safety* (Table 7).

**Table 7:** Factors loading in each item

***Items***	***1***	***2***	***3***	***4***	***5***	***6***	***7***	***8***
A1. People support one another in this facility	0.57							
A3. When a lot of work needs to be done quickly, we work together as a team to get the work done	0.64							
A4. In facility, people treat each other with respect	0.50							
A11. When one area in this unit gets really busy, others help out	0.66							
B2. Manager says a good word when he/she sees a job done according to established		0.63						
B3. Whenever pressure builds up, my supervisor/manager wants us to work faster, even if it means taking shortcuts		0.50				0.44		
B4. My supervisor/manager overlooks patient safety problems that happen over and over						0.58		
A6. We are actively doing things to improve patient safety.							0.47	
A13. After we make changes to improve patient safety, we evaluate their effectiveness.							0.58	
F1. Hospital management provides a work climate that promotes patient safety.	0.65							
F8. The actions of hospital management show that patient safety is a top priority.	0.70							
F9. Hospital management seems interested in patient safety only after an adverse event happens	0.56							
A10. It is just by chance that more serious mistakes don't happen around here.	0.53							
A17. We had patient safety problems in this unit.	0.44							
A18. Our procedures and systems are good at preventing errors from happening.	0.59							
C1. We are given feedback about changes put into place based on event reports.	0.59							
C3. We are informed about errors that happen in this unit.		**−0.54**						
C5. In this unit, we discuss ways to prevent errors from happening again.	0.41							0.43
C2. Staff will freely speak up if they see something that may negatively affect patient care.	0.57							
C4. Staffs are afraid to ask questions when something does not seem right.	0.48							
C6. Staffs feel free to question the decisions or actions of those with more authority.	0.55							
A8. Staff feel like their mistakes are held against them.			0.42					
A12. When an event is reported, it feels like the person is being written up, not the problem.			0.66					
A16. Staff worry that mistakes they make are kept in their personnel file.			0.59					
F4. There is good cooperation among hospital units that need to work together.	0.66							
A2. We have enough staff to handle the workload.	0.68							
A5. Staffs in this unit work longer hours than is best for patient care.		**−0.47**			0.41			
A7. We use more agency/temporary staff than is best for patient care.	**−0.41**			0.43				
A14. We work in “crisis mode” trying to do too much, too quickly.	0.40							

## Discussion

Safety culture originated from high reliability organizations (HROs) in the last several decades, which has gained much attention in health care fields to promote patient safety recently both in individual work units or hospitals [23]. This has improved since the Hospital Survey on Patient Safety Scale (HSPSC) was introduced by the Agency for Healthcare Research and Quality (AHRQ). The HSPSC survey has been translated into 24 languages in 45 countries to measure patient safety culture in their own healthcare organizations [18,21].

In our study, we used revised HSPSC to measure patient safety culture in China. A total of 1,500 of questionnaires were distributed to 32 hospitals in 15 cities across China of which 1,160 respondents were eligible. The response rate was 77%, which was similar to the study implemented in Taiwan [10]. The overall positive response rate for 29 items was acceptable which was higher than the other two studies conducted in Taiwan by Chen and the mainland of China by Zhu [10,19]. Comparing with the two studies by Chen 2010 and by Zhu 2012, we found that the participants surveyed by the study in Taiwan was a light different from our study regarding to the included participants that 29% of the respondents were physicians, 60% were nurses, and 10% were administrators, while there was no administrators included in our study. There was also a time difference between the two studies .The research in Taiwan was conducted from January 2006 to February 2008 which was three years earlier than that of our study. The results showed the dimension that received the highest positive rate was ‘Teamwork within units’, and the lowest percentage of positive responses was ‘Staffing’ which is also similar to our study. There was also a difference from the study by Zhu 2012 [19] from our study regarding to the purpose that the study by Zhu 2012 focused on assessing the appropriateness of existing safety culture questionnaires used in the USA and Japan for Chinese respondents. In this study, the authors identified new items and domains suitable to Chinese hospitals. Eight new items and three additional dimensions were identified addressing staff training, mentoring of new hires, compliance with rules and procedures etc. The results from both studies recognized that adequate staff is crucial for patient safety and is one of the biggest challenges which is consistent with the results from our study.

The result showed that the positive response rate for each item ranged from 36% to 89%. The dimensions that received the highest positive response rate were *Teamwork Within Units,* while the dimensions for the lowest positive response rate was *Staffing.* Moreover, the positive response rate of five dimensions*: Teamwork Within Units, Organization Learning-continuous Improvement, Communication Openness, Non-punitive Response to Errors and Teamwork Across Units* in Chinese hospitals (84%, 88%, 65%, 60%, and 66%, respectively) was higher than that of US hospitals (80%, 75%, 72%, 62%, 44% and 58%, respectively). The results may imply one of the core values of Chinese culture is prioritizing harmony. The Chinese are warm and willing to help others and place relatively more emphasis on cooperation and learning [24-26]. The positive response rate of four dimensions of *Staffing, Feedback & Communication About Errors*, *overall Perception of Patient Safety and Management Support* for *Patient Safety* (45%, 50%, 55% and 69%, respectively) was lower than that of US study (56%, 64% 66% and 72%, respectively). The results suggested many health care workers in Chinese setting shy away from discussing or reporting adverse events, asking questions or challenging those with more authority even when they disagree [19]. The lowest percentage of positive response rate was *“Staffing*”, which means that most of the respondents felt that staff allocation is not adequate to handle patient safety related workload, especially in some comprehensive hospitals where there is a greater ratio of patients to staff. A similar finding was reported by Hellings and the study conducted in Taiwan and China [10,17,19].

The perception of patient safety culture was different in different environments, for example working units, professionals and so on [12]. The results showed that the positive response numbers of nurses regarding patient safety culture was higher than that of physicians in our study. Nurses spent more time in contacting and communicating with patients [22,27], so they had more opportunity to deal with patient safety issues. In China there is a strict professional training about patient safety in clinical practice for nurses which may account for this higher positive response. In addition, our study found that the positive response rate of physicians with a high qualification level in dimensions of *Overall Perception of Patient Safety* and *Feedback & Communication About Errors* was higher than that of physicians with a low qualification level, while in some dimension such as *Teamwork Within Units, Management Support For Patient Safety, Communication Openness, Teamwork Across, Units and Non-punitive Response to Errors* was lower than that of physicians with a low qualification. The results were consistent with two studies by Said B. Turkey in 2009 and 2012 [13,28].

Patient safety in health care system has gained much attention since the Institute of Medicine (IOM) reported the publication of To Err Is Human: Building a Safer Health System. As high as 44 000–98 000 of the people died from medical errors annually in US, what is alarming is the number of deaths, permanent disability and avoidable injuries that has resulted from the high incidence of medical error. Patient safety issues in developing countries are also questionable. E.g. Relevant studies in China showed that a total of 200,000 patients died from drug adverse every year, and 10%-30% of inpatients suffered from drug adverse reactions each year [29]. Chinese Hospital Association (CHA) estimated that adverse events affect 1.6 ~ 7.6 million hospitalizations annually in Chinese hospitals [30]. The HSOPSC survey results in our study demonstrated that the hospitals and health care organizations in China should develop strategies to improve health quality and ensure patient safety. These strategies include: providing training and education on patient safety for health care workers in different levels (undergraduate education, continuing education, lectures and meetings); allocating enough staff and adequate workload; developing and fostering patient safety culture especially in the form of a non-punitive culture, creating an open communication atmosphere for reporting medical errors and speaking up when any problem arises.

### Reliability and validity

Our result showed that internal consistency reliability was acceptable in China (Cronbach’s α = 0.84), eight factors were drawn, which could explain 60% variance. The results were less satisfactory than those of the US [31]. However the results were almost similar to the study conducted in Norway [12]. Three reasons could account for this. Firstly, scale should not be translated and applied in another setting of a different cultural context directly [11,19]; secondly, culture of organization leadership, policy belief and management pattern were diverse between the US and China; thirdly, 13 original items were omitted from the report, therefore the, reliability and validity could contribute to the change.

### Advantages and limitations

This is the first kind of study that was conducted in China in different cities and hospitals with different health care workers. This is different from other studies published in China that only focused on nurses or assessment only on the scale of HSPSC [19]. The results of this study may provide some evidence to help relevant Chinese decision makers develop effective strategies on improving the quality of health care to ensure patient safety. However, our study also has some limitations. Firstly, thirteen original items were deleted that might influence the framework of the patient safety culture survey. Secondly, there were very few respondents from the hospital management level, which may not reflect the whole picture of patient safety culture in China.

## Conclusion

The results demonstrated that amongst the health care workers surveyed in China there was a positive attitude towards patient safety culture in their organizations. Different position, qualification and work units may have different responses for different dimensions or items. The questionnaire used in our study was acceptable according to HSPSC (version 2010).

## Competing interests

The authors declared that they have no conflict of interests.

## Authors’ contributions

YLN and XYM contributed equally to this study. MMZ conceptualized and designed the study. YLN, CH and JL, SH H did data collection and input. XYM checked and review data. YLN performed the data analysis and XYM contributed to the interpretation of the data. MMZ and XYM contributed to and revised the manuscript critically for intellectual content. All authors read and approved the final draft.

## Pre-publication history

The pre-publication history for this paper can be accessed here:

http://www.biomedcentral.com/1472-6963/13/228/prepub
